# Evaluating levels of knowledge and food safety practices among food handlers in the Southern part of Italy

**DOI:** 10.1016/j.heliyon.2024.e30722

**Published:** 2024-05-04

**Authors:** Francesca Licata, Giorgia Della Polla, Natascia Costantino, Concetta Paola Pelullo, Aida Bianco

**Affiliations:** a Department of Health Sciences, School of Medicine, University of Catanzaro “Magna Græcia”, Viale Europa, Catanzaro, Italy; b Health Direction, Teaching Hospital, University of Campania “Luigi Vanvitelli”, Naples, Italy; c Department of Movement Sciences and Wellbeing, University of Naples “Parthenope”, Naples, Italy; d Department of Medical and Surgical Sciences, School of Medicine, University of Catanzaro “Magna Græcia”, Viale Europa, Catanzaro, Italy

**Keywords:** Food handlers, Food handling practices, Food safety, Foodborne illnesses, Italy, Knowlwdge

## Abstract

The purpose of the present study is to assess the extent of knowledge and food safety practices among food handlers (FHs) to prevent food risks. This cross-sectional study was conducted between July 2021 and February 2022 in a random sample of FHs working in three regions of the Southern part of Italy. A two-stage cluster random sampling strategy was used to select FHs working at food businesses in the Regions. Data were collected through an anonymous self-administered questionnaire that consisted of 33 questions grouped into five sections to gather sociodemographic and professional characteristics, knowledge about foodborne illnesses (FBIs) and food safety, adherence to proper food handling practices and sources of information.

The overall median knowledge score was 8 (interquartile range 6–9), but only 2.2 % of the respondents answered all 12 statements correctly. Among the recruited FHs, 71.2 % and 65.4 % reported always keeping raw and cooked food separate and using different utensils while handling raw and cooked foods, respectively. With respect to the use of personal protective equipments, 79.3 % and 67.6 % stated always wear work clothing and hair restrain (e.g., hats, hairnets), respectively. Just 20.9 % of the FHs properly defrosted food (i.e., in the fridge) and 39.9 % used food warmers for keeping food at least at 65 °C while waiting for service.

The findings highlighted poor knowledge concerning the ideal temperatures for cooking, holding and storing foods, exacerbated by poor personal and hand hygiene, certain factors associated with the spread of foodborne pathogens.

## 1 Introduction

Foodborne illnesses (FBIs) continue to be a major public health concern despite being largely preventable [1]. The majority of foodborne disease cases reported in EU nations in 2021 occurred in restaurants [2]. It is generally acknowledged that poor food preparation and food service techniques are strongly correlated with poor microbiological quality of served foods [3]. Numerous foodborne outbreaks are caused by improper food handling during food production processes (e.g., cooking, storing, and serving food) [4]. Governments all over the world have enacted food safety laws to safeguard consumers' health and ensure responsible practices in the food industry. One of the five goals outlined in the Codex Alimentarius Commission's 2020–2025 Strategic Plan, which was released in 2019, is the development of regulations based on evidence-based and risk analysis principles [5]. Food handlers (FHs) have the potential to contaminate food by using poor hand hygiene, not keeping surfaces and equipment clean, or handling and preparing food incorrectly. Hence, FH participation is crucial in FBIs prevention. FHs are in charge of making sure that the regulations of the food law are followed and, ultimately, protecting consumers' health. FHs are required to ensure the product's quality to the best of their abilities. In order to guarantee food quality and prevent the spread of FBIs, they must comply with food safety protocols. Therefore, FHs' staff members should be knowledgeable about food contaminants, FBIs, hygienic food handling, and good production techniques. In Italy, according to the current legislation, every food business is required to adhere to a set of fundamental regulations and the most popular food safety system is Hazard Analysis and Critical Control Points (HACCP) [6], a systematic procedure for self-controlling health risks that prevents any possibility of contamination or alteration in the characteristics of the product. Moreover, managers of the food sector, FHs, and also personnel involved in the food process but not in direct contact with food must have a certificate to prove they have received the required education in food safety and hygiene. Indeed, FHs need appropriate food safety skills and knowledge commensurate with their work activities. Therefore, the purpose of the present study is to assess the extent of knowledge and food safety practices among FHs, to prevent food risks.

## 2 Materials and methods

### 2.1 Study design and setting

The cross-sectional study was conducted between July 2021 and February 2022 in a random sample of FHs working in three regions (i.e., Calabria, Campania, and Sicily) of the Southern part of Italy. The reporting of the present study complies with the Strengthening the Reporting of Observational Studies in Epidemiology (STROBE) standards for reporting observational studies [7].

### 2.2 Study population and data collection

The criteria to be eligible for participation to this study were: (1) age ≥18 years, (2) working in food business as FH. The exclusion criteria were: 1) not being able to understand, speak, and read Italian and 2) refusing to sign the informed consent form. A two-stage cluster random sampling strategy was used. Each Region was divided into cities and one city was randomly selected within each Region. Then, from a publicly available frame [8], 30 food businesses (clusters) were randomly selected within each city. All FHs working at the selected food business were approached and invited to participate in the study. All participants were assured that there were no identifiers that linked responders to the questionnaire and that they could withdraw their participation at any moment. The food business owner was not involved in the recruitment, data collection, or consent process for any of the participants. No payment or incentive were given to participants in exchange for their participation.

### 2.3 Questionnaire

Data were collected through an anonymous self-administered questionnaire based on a comprehensive literature review [[9], [10], [11], [12], [13], [14]]. The questionnaire was pre-tested in a random sample of 15 FHs not included in the final sample. This phase provided the research team with feedback that confirmed adequate comprehension of the questions. Only minor refinements were made to improve flow. The final questionnaire was designed to be completed within 15 min and it consisted of 33 questions grouped into five sections. The first section collected sociodemographic (i.e., gender, age, marital status, education level) and professional characteristics (i.e., professional category and duration of work activity). The second section was designed to evaluate the FHs' knowledge about FBIs and food safety (12 questions with a “true/false/don't know” response format). The third section (12 questions closed-ended with multiple answers) explored the adherence to proper food handling practices to prevent food cross-contamination, personal protective equipment (PPE) use, hand hygiene (HH) and practices to keep food at safe temperatures. The last section investigated sources of information about food safety, satisfaction with these sources, and the need to receive further information on FBIs.

The Calabria Centre Local Human Research Ethics Committee (ID No. 271/2021/07/15) approved the study protocol.

### 2.4 Statistical analysis

All collected variables were summarized by means and standard deviations (SD) when normally distributed. Medians and interquartile ranges (IQR) were used in cases of deviations from normality. The skewness of the variables was estimated by Shapiro-Wilk tests. Categorical variables were expressed as percentages. The following outcomes of interest were investigated: knowledge score about FBIs and food safety (Model 1), adherence to practices preventing food cross-contamination (Model 2), personal hygiene practices (Model 3) and appropriate management of temperatures for food safety (Model 4). The knowledge score was computed by assigning one point for each right response and summing the scores for each statement (range 0–12), and an ordinal regression model was built to analyze the independent association of explanatory variables and this outcome of interest (Model 1). In Model 2, the outcome was identified by having always kept raw and cooked food separate and having always handled raw and cooked foods using different utensils versus all others. In Model 3, FHs were divided into those who implemented food safety practices (i.e., having always worn work clothing and hair restrain while cooking and having always washed their hands before starting work and touching ready-to-eat-food, and after touching raw food, blowing the nose, using the toilet, handling potentially contaminated raw food) versus all other. In Model 4, respondents were divided into those who appropriately kept food at safe temperatures (i.e., those who always use blast chillers for cooling food, who always use food warmers to keep aliment at 65 °C or above to preserve cooked foods while waiting for service and who properly defrost food in the fridge) versus all other. Multiple logistic regression models were developed to investigate the independent association of explanatory variables and the outcomes of interest of Models from 2 to 4. The following explanatory variables were included in all Models: gender (male = 0; female = 1), education level (secondary school or lower = 0; high school or higher = 1), marital status (married/cohabitant = 0; others = 1), nationality (Italian = 0; foreign = 1), duration of working activity, in years (continuous) and actively handling food (i.e., chefs, sous chefs, cooks, kitchen assistants) (no = 0; yes = 1). The knowledge score (continuous) was included in Models 2, 3 and 4. The practices to keep food at safe temperatures (no = 0; yes = 1) was included in Models 2 and 3. The variable proper personal hygiene practices (no = 0; yes = 1) was included in Models 2 and 4. Adherence to practices preventing food cross-contamination was included in Models 3 and 4.

The statistical significance level was set at a p-value <0.05. Adjusted odds ratios (ORs) and 95 % confidence intervals (CIs) were calculated. Statistical analysis was performed using STATA software program, version 18 [15].

## 3 Results

### 3.1 Sociodemographic and professional characteristics

All selected food businesses agreed to participate in the study. Of the 638 FHs approached, 454 (71.2 %) completed the questionnaire. More than half were male (52.5 %), and the mean age was 38.8 years (SD ± 12 years). The majority held high school qualification (74.2 %) and more than half (54.9 %) was married or cohabitant. The average duration of working activity was 13.5 years (SD ± 10.8 years) and almost one-third (31.1 %) were actively handling food.

### 3.2 Knowledge related to FBIs and food safety

**Fig. 1:**
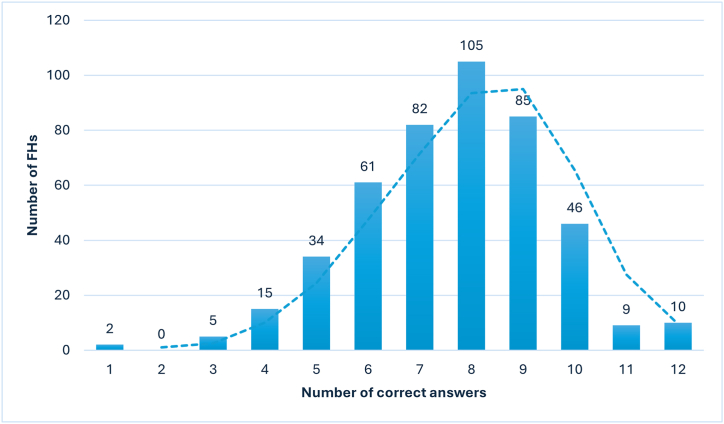
Absolute frequency of correct answers to the knowledge statements about FBIs and food safety.

Table 1 presents the answers to the statements about knowledge on FBIs and food safety. Most of the participants knew that foods must be well cooked to prevent FBIs (90.3 %) and that the proper refrigerator storage temperature of creamy and dairy products is between 2 and 4 °C (86.6 %) and of frozen foods ≤ −18 °C (79.8 %). Less than one-third (30.2 %) acknowledged that cooked food must be cooled quickly if it is not consumed in a short period of time and 80.4 % wrongly recognized that food should be kept at room temperature until its internal temperature is cooler than 10 °C. Moreover, almost one-fifth (17 %) were unknowledgeable that food cross-contamination (e.g., by using the same utensils for cooked and raw foods) could lead to FBIs and even 74 % of FHs did not identify the five steps to properly wash their hands. The overall median knowledge score was 8 (IQR 6–9), but only 2.2 % of the respondents answered all the 12 statements correctly (Fig. 1). The results of the ordinal regression analysis showed that the knowledge score was significantly higher among FHs with more years in practice (OR: 1.02, 95 % CI: 1.01–1.04) and actively handling food (OR: 1.47, 95 % CI: 1.01–2.13).

**Table 1:** Knowledge related to food-borne diseases.

Statements (454 respondents)	Correct answers	
	N	%
Foods must be well cooked to prevent foodborne diseases (True)	410	90.3
Cooked ready-to-eat foods must be maintained at a temperature of at least 60 °C until served (True)	263	57.9
Cross-contamination is a cause of foodborne infections (True)	377	83
The storage temperature of creamy and dairy products in the refrigerator is between 2 and 4 °C (True)	393	86.6
The storage temperature of frozen foods is ≤ −18° (True)	362	79.8
Blast chiller can lower the temperature of cooked food (True)	329	72.4
Work clothes and personal clothes have to be stored separately (True)	419	92.3
Work clothes should be dark in colour in order to camouflage stains (False)	381	83.9
Cooked food must be cooled quickly if it is not consumed in a short period of time (True)	137	30.2
Food must be kept at room temperature before being refrigerated by decreasing its internal temperature from 60 to 10° (False)	89	19.6
Knowledge of the five steps to wash hands the right way^a^	118	26
Adolescents, children, elderly, pregnant, and immunocompromised people are at higher risk of developing a severe foodborne disease than the general population^b^	57	12.6

a Wet your hands with clean, running water (around 45 °C). Apply soap and lather the hands by rubbing them together with the soap (lather the backs of the hands, between the fingers, and under the nails). Scrub the hands for at least 20 s/Brush the nails with a special brush. Rinse the hands well under clean, running water. Dry the hands using a clean towel or an air dryer.

b Answers were considered correct if the participant indicated all the people at higher risk of developing severe foodborne disease than the general population.

### 3.3 Behaviors regarding food handling practices

Food handling practices are displayed in Table 3. Regarding practices to prevent food cross-contamination, 71.2 % and 65.4 % of FHs reported always keeping raw and cooked food separate and using different utensils while handling raw and cooked foods, respectively; three-fifths (59 %) affirmed always doing both practices. The logistic regression model evaluating adherence to practices preventing food cross-contamination showed that each one-point increase in the knowledge score led to a 37 % increase (OR: 1.37; 95 % CI:1.19–1.57) in the odds of adherence to preventive practices. Furthermore, the FHs’ adherence to these preventative practices positively and significantly correlated with proper personal hygiene practices (OR: 4.36; 95 % CI:2.68–7.01), keeping food at safe temperatures (OR: 13.91; 95 % CI: 1.66–116.55), foreign nationality (OR: 0.27; 95 % CI:0.11–0.71) and not needing further information on FBIs (OR: 0.61; 95 % CI: 0.39–0.97) (Model 2 in Table 2).

**Table 2:** Results of the regression models for potential determinants of different outcomes of interest.

	Model 1		Model 2		Model 3		Model 4	
	Knowledge score about FBIs and food safety		Adherence to practices preventing food cross-contamination		Proper personal hygiene		Practices to keep food at safe temperatures	
	*Log likelihood = -815.277713;* *Prob > chi2 < 0.000; Obs = 426*		*Log likelihood = -226.87739;* *Prob > chi2 < 0.001; Obs = 432*		*Log likelihood = -235.03341;* *Prob > chi2 < 0.001; Obs = 432*		*Log likelihood = −90.663868;* *Prob > chi2 < 0.001; Obs = 432*	
	OR	95 % CI	OR	95 % CI	OR	95 % CI	OR	95 % CI
Gender (Male as reference)	0.91	0.64–1.30	1.39	0.86–2.25	0.98	0.61–1.57	1.39	0.35–1.73
Education level **(**Secondary school or lower as reference)	1.03	0.66–1.62	0.85	0.44–1.65	0.74	0.40–1.37	0.34	0.14–0.84
Marital status (Married as reference)	0.77	0.53–1.11	0.92	0.57–1.49	1.06	0.66–1.71	0.88	0.38–2.02
Nationality (Italian as reference)	0.69	0.36–1.34	0.27	0.11–0.71	1.91	0.78–4.70	1.44	0.25–8.35
Duration of working activity in years, continuous	1.02	1.01–1.04	1.01	0.98–1.03	0.99	0.97–1.01	0.99	0.96–1.04
Actively handling food (No as reference)	1.47	1.01–2.13	0.95	0.58–1.57	1.11	0.68–1.81	1.55	0.69–3.44
Need for further information on FBIs (No as reference)	0.74	0.52–1.04	0.61	0.39–0.97	0.70	0.44–1.10	0.82	0.34–2.03
Knowledge score about FBIs and food safety, continuous	–		1.37	1.19–1.58	1.41	1.21–1.63	1.59	1.22–2.09
Adherence to practices that prevent food cross-contamination (No as reference)	–		–		4.25	2.61–6.90	9.71	1.24–76.32
Proper personal hygiene (No as reference)	–		4.36	2.68–7.09	–		4.04	1.31–12.50
Practices to keep food at safe temperatures (No as reference)	–		13.91	1.66–116.54	4.54	1.43–14.43	–	

FBIs: foodborne illnesses.

**Table 3:** Food handling practices.

Statements (454 respondents)	Never		Rarely		Occasionally		Often		Always	
	N	%	N	%	N	%	N	%	N	%
Check the integrity of packages before using them	–		2	0.4	15	3.3	67	14.8	**370**	**81.5**
Check food products expiration date	1	0.2	1	0.2	26	5.7	68	15	**358**	**78.9**
Keep raw and cooked food separate	11	2.4	26	5.7	45	9.9	49	10.8	**323**	**71.2**
Handle raw and cooked foods using different utensils	12	2.6	21	4.6	72	15.9	52	11.5	**297**	**65.4**
Use work clothing while cooking.	5	1.1	3	0.6	33	7.3	53	11.7	**360**	**79.3**
Use of hair restrains (e.g. hat/hairnets)	24	5.3	14	3.1	56	12.3	53	11.7	**307**	**67.6**
Use thermometer for at least 5–10 min to make sure that internal temperature of food has reached ≥75 °C	71	15.7	31	6.8	134	29.5	41	9	**177**	**39**
Use food warmer that keeps food at least at 65 °C to preserve cooked perishable foods while waiting for service	79	17.4	37	8.1	109	24	48	10.6	**181**	**39.9**
Use blast chillers for cooling food	74	16.3	31	6.9	109	24	46	10.1	**194**	**42.7**

	Yes	
	N	%
Occasions in which you wash your hands:^a^		
Before starting work	409	90.1
Before touching ready-to-eat-food	376	82.8
After touching raw food	331	72.9
After blowing your nose	391	86.1
After using the toilet	426	93.8
After handling potentially contaminated raw food	362	76.7
All the above	276	60.8
Properly disinfecting and covering the wounds with a blue-plaster wearing gloves.	414	93.2
Properly defrosting frozen food (i.e., in the fridge)	92	20.9

Number and percentages referring to correct answers are in bold.

a Multiple responses allowed.

**Fig. 2:**
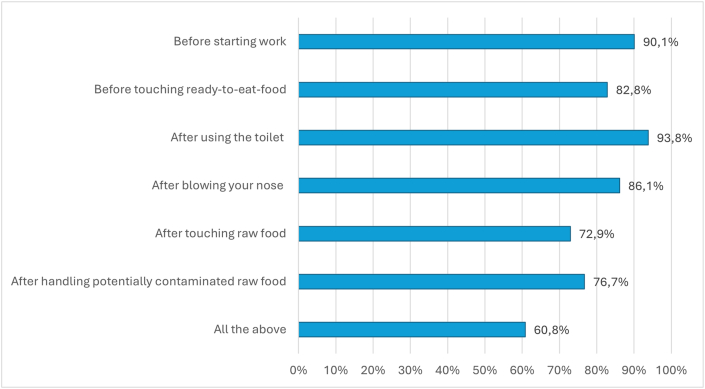
Relative frequency of FHs that wash their hands in each of the suggested occasions.

With respect to the use of PPEs, 79.3 % and 67.6 % stated they always wear work clothing and hair restrain (e.g., hats, hairnets), respectively. Regarding the occasions in which they wash their hands, 27.7 % and 23.3 % of FHs stated that they did not wash their hands after touching raw food and handling potentially contaminated raw food, respectively; 60.8 % washed their hands in all the selected occasions (Fig. 2). The results of Model 3 in Table 2 showed that a one-point increase in the knowledge score led to a 41 % increase in the odds of proper personal hygiene practices (OR: 1.41; 95 % CI: 1.22–1.62), that significantly increased also among those who keep food at ideal temperatures (OR: 4.25; 95 % CI: 2.61–6.90) and adhere to practices preventing food cross-contamination (OR: 4.54; 95 % CI: 1.43–14.43).

Just 20.9 % of the FHs properly defrosted food (i.e., in the fridge). About two-fifths of the sampled FHs declared using blast chillers for cooling food (42.7 %) and food warmers for keeping food at least at 65 °C while waiting for service (39.9 %). Of the participants, 8.2 % appropriately managed temperatures for food safety and that was more likely among those FHs who had a good knowledge score about FBIs and food safety (OR: 1.59; 95 % CI: 1.22–2.09), among those who implemented proper personal hygiene practices (OR: 4.04; 95 % CI: 1.31–12.50) and measures preventing food cross-contamination (OR: 9.71; 95 % CI:1.24–76.32) (Model 4 in Table 2).

### 3.4 Sources of information

**Fig. 3:**
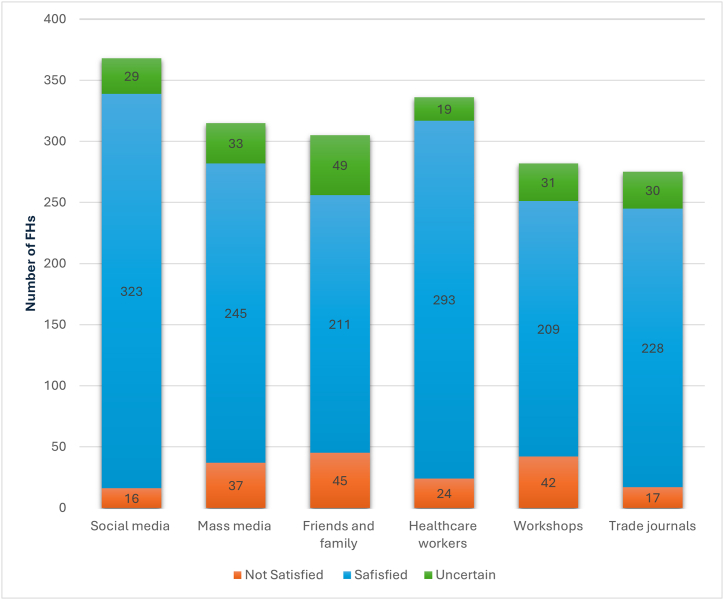
Types of information sources about food safety and levels of satisfaction with the information received.

Among the sources of information about food safety, FHs most frequently mentioned social media (78.9 %) and mass media (78.2 %), followed by friends and family (75.8 %), and healthcare workers (73.6 %), declaring to be most satisfied with the information provided by social media (90.2 %) and healthcare workers (87.7 %) (Fig. 3). Less than half of the sample (44.3 %) reported the need for more information about FBIs.

## 4 Discussion

The present research provides up-to-date data about knowledge and practice gaps related to temperature for food safety, personal hygiene, and food handling procedures among FHs. The role of FHs to achieve the public health goal of reducing FBIs is a pivotal one. Therefore, it is important to determine gaps in food safety framework to guide effective educational and behavioral interventions among this professional group.

A key result of the study states that FHs in the sample had insufficient knowledge of the ideal temperature for holding food. Indeed, they neglected the importance of keeping cooked food at a minimum temperature of 60 °C while waiting for serving. Similarly, a noticeable proportion of FHs ignored that cooked food, if it is not consumed in a short period of time (within 2 hrs), must be cooled as quickly as possible to prevent proliferation of pathogen microorganisms and/or formation of toxins [16]. Furthermore, a vast majority of the sample assumed that, before being refrigerated, food must be kept at room temperature to decrease its internal temperature. All these figures are points of concern because one of the main causes of FBIs is leaving food out at an inadequate temperature [17]. Controlling temperature is one of the most influential factors to preserve cooked food and ensure their quality and safety. At low temperatures, pathogen microorganisms grow very slowly, at moderate temperatures (danger zone) they multiply quickly, and at high temperatures they are destroyed. It should also be pointed out that lack of knowledge about the food cold chain was also highlighted by the fact that most of the enrolled FHs did not safely defrost frozen food (just 20.9 % properly thaw frozen foods in the fridge). The risk of FBIs increases because of improper thawing methods, which can promote the growth of pathogen microorganisms and cause spoilage [17]. Along with that, if food is not properly thawed, it can lead to uneven cooking and food safety issues [18]. All the above-mentioned issues underscore the need to reinforce FHs' awareness about all the steps that must be taken at each point in the food chain where hazards can occur, such as proper storage and reheating techniques that should be followed to ensure that the food remains safe for consumption. It is widely known that control of the temperature is one of the most important critical control points in ensuring food safety and preventing FBIs that requires implementation of measures to prevent, eliminate, or reduce the food safety hazard [19].

The study's results referring to personal hygiene measures were even more worrying, both in knowledge and practice domains. Indeed, more than half of the sample did not know all the steps to properly wash their hands and almost 30 % stated not to do so after handling or preparing raw food. One of the most crucial components of personal hygiene strategies to prevent food contamination is hand hygiene that has frequently been a central factor in foodborne outbreaks [20,21]. Routine hand hygiene easily removes transient microorganisms and can prevent the spread of harmful microorganisms from hands to food, and ultimately to consumers [22]. It is important for FHs to wash their hands frequently and thoroughly, following established guidelines and using appropriate products, especially before handling food or after touching any potentially contaminated surface. Evidence exists indicating that if each FH regularly washed their hands, 1 million deaths could be avoided each year [23]. It is well-known that all FHs who have direct contact with food must wear hair restraints, such as hairnets, beard nets, and caps, that fully cover all exposed body hair, and the figure that one-third of the sample declared that they did not always use hair restraints is of concern. High standards of personal hygiene, including the appropriate use of PPE, can control and remarkably reduce the risks of the spread of pathogen microorganisms to everything FHs touch, including ready-to-eat food. One of the keys to the prevention of food contamination by FHs is the ability to maintain high standards of personal hygiene. To do this FHs should have ongoing training in the importance of personal hygiene and hand washing, together with regular assessment of knowledge and practices.

Another key finding of this study is that a sizeable percentage of FHs failed to check the expiration date or the integrity of packages before using them. Consuming expired foods can increase the likelihood of FBIs which can lead to serious health consequences for the consumers, especially for frail populations [24]. Many outbreaks of FBIs could be avoided if certain precautions are taken, such as adequate practices for handling food, coupled with proper packaging and storage [25]. FHs are required to check the integrity of packages and the expiration date on them as well as to keep raw and cooked food separated and to use different utensils for them. Nonetheless, risky habits like mixing raw and cooked food together or not using different cooking and serving utensils for raw and cooked food preparation still pose a threat to food safety and, ultimately, to public health. As shown from the practice assessment carried out in the present study, important practices to prevent cross-contamination are lacking in a large portion of the sample. The risk of cross-contamination is, therefore, high in similar framework: microorganisms can be transferred directly when raw food touches or drips onto ready-to-eat foods or they can be indirectly transmitted by contaminated hands, tools, and work surfaces [26].

Undoubtedly, cross-contamination is a major concern in food preparation. It is critical that FHs can recognize potential hazards and put the necessary precautions in place to avoid contamination and proliferation of the pathogens in the food. Consumers may ultimately benefit from a safer and healthier food supply chain because of this. The food supply chain can be kept safe and wholesome for all consumers by enforcing strict regulations and inspections and educating FHs about proper food handling techniques. The findings of the present study shed light upon the FHs' habits that are most likely to lead to FBIs and could be helpful to design local hygiene training programs. Although knowledge by itself could be insufficient to encourage preventive behaviors [27], evidence from the literature indicates that the effectiveness of food hygiene training as a tool for raising food safety standards is limited by a lack of knowledge about the elements that contribute to successful results [28]. Programs promoting good hygiene, for example, have the potential to change behavior and have a higher chance of success if they are based on local research and employ frequent, sustained communication through channels that are appropriate to the local environment [29].

### 4.1 Limitations

The present study has some limitations that should be acknowledged to appreciate its findings. First, the study sample was limited to FHs in three regions, which might not represent all Italian FHs. However, the findings of the study may be representative at least for the Regions of Southern Italy. Second, the data were self-reported increasing the possibility of desirability bias; but direct observation was not feasible due to the expense involved and the risk of producing observation bias. Nonetheless, assurance of anonymity and confidentiality of the data in the survey minimizes the probability of this bias. Participation bias could even lead to a possible overestimation of knowledge, as the better-informed FHs might have been more likely to participate. However, the level of knowledge among recruited FHs suggests that this bias had no substantial effect on the results.

## 5 Conclusions

Even with these potential limitations, the present study publicizes reliable baseline data about the potential mechanisms of food contamination during processing and manufacturing of food. In the authors’ opinion, the FHs' role on the food safety journey is a pivotal one to achieve the public health goal of reducing FBIs to the fullest extent possible. The figure that FHs exhibited poor knowledge concerning the temperatures for cooking, holding and storing foods, coupled with poor personal and hand hygiene, has been a matter of serious concern since both represent certain factors associated with the spread of foodborne pathogens. Food safety training and the provision of hygienic, supportive work environments are essential to ensuring a food safety culture. Additional investigations are required into new fields like course content, training location, course length, and refresher training [28]. To produce results that are worthwhile, such research must be carefully planned, well-designed, and supported by solid reliable baseline data [30].

## Ethics statement

The Calabria Centre Local Human Research Ethics Committee (ID No. 271/2021/07/15) approved the study protocol.

## Funding

This research did not receive any specific grant from funding agencies in the public, commercial, or not-for-profit sectors.

## Data availability statement

The anonymized data collected in this study was deposited in Mendeley Data repository (https://doi.org/10.17632/vtvnz52wgs.1).

## CRediT authorship contribution statement

**Francesca Licata:** Validation, Visualization, Writing – original draft, Conceptualization, Data curation, Formal analysis, Investigation. **Giorgia Della Polla:** Investigation, Writing – original draft. **Natascia Costantino:** Conceptualization, Data curation, Investigation, Writing – original draft. **Concetta Paola Pelullo:** Investigation, Writing – original draft. **Aida Bianco:** Project administration, Resources, Supervision, Conceptualization, Investigation, Writing – review & editing, Methodology.

## Declaration of competing interest

The authors declare that they have no known competing financial interests or personal relationships that could have appeared to influence the work reported in this paper.
